# Sewage sludge as substrate in *Schinus terebinthifolia* raddi seedlings commercial production

**DOI:** 10.1038/s41598-022-21314-0

**Published:** 2022-10-14

**Authors:** Jorge Makhlouta Alonso, Renato Nunes Pereira, Elton Luis da Silva Abel, Marjorie Ochoski, Gilsonley Lopes dos Santos, Alan Henrique Marques de Abreu

**Affiliations:** 1 Lato Sensu Postgraduate Program in Applied Statistics, Mathematics Department, Institute of Exact Sciences, Universidade Federal Rural Do Rio de Janeiro, BR 465, km 07, Seropédica, RJ 23890-000 Brazil; 2 Mathematics Department, Institute of Exact Sciences, Universidade Federal Rural Do Rio de Janeiro, BR 465, km 07, Seropédica, RJ 23890-000 Brazil; 3 Companhia Estadual de Águas e Esgotos – CEDAE, BR 465, km 19,5 ETA Guandu - CEP: 26.298-000, Nova Iguaçu, RJ Brazil; 4 Silviculture Department, Institute of Forestry, Universidade Federal Rural Do Rio de Janeiro, BR 465, km 07, Seropédica, RJ 23890-000 Brazil; 5 Stricto Sensu Postgraduate Program in Environmental and Forest Sciences, Institute of Forestry, Universidade Federal Rural Do Rio de Janeiro, BR 465, km 07, Seropédica, RJ 23890-000 Brazil

**Keywords:** Plant sciences, Plant breeding, Plant domestication, Forestry, Environmental sciences

## Abstract

*Schinus terebinthifolia* Raddi is a species with several potential uses; selecting the proper substrate and fertilizer rate can be vital for seedling production in a nursery environment. This study aims to evaluate two substrates, namely: (i) sewage sludge (SS) from Ilha WWTP; (ii) a commercial substrate (CS) made of organic materials (mainly sphagnum peat). Increasing rates of controlled-release fertilizer – CRF (0, 3, 6, and 12 kg m^−3^) were applied. The experiment was completely randomized with a factorial 2 × 4 scheme (substrates × rates). The seedlings’ growth, biomass, and quality were evaluated. The treatments were compared by Tukey test and regression analysis, where linear, quadratic, and cubic models were considered. Principal components analysis (PCA) and cluster analysis were performed. The CRF rates showed non-significant effects for most of the investigated variables in the SS substrate. In the CS, a 7.8 kg m^−3^ rate of CRF showed the best growth performance. The multivariate analysis of the morphological parameters proved suitable as a complementary approach to evaluate the seedlings’ quality. Seedlings reached recommended values for height, diameter, and quality in the 100% SS substrate without chemical fertilizers; thus, *Schinus terebinthifolia* production in the SS from Ilha is recommended. Besides the growth advantage, the SS can promote nursery cost savings with commercial substrates and chemical fertilizers.

## Introduction

*Schinus terebinthifolia* Raddi (rose pepper) is an arboreal shrub species naturally occurring in the tropical and subtropical Atlantic Forest of South America^1^. The species has several uses that stimulate its propagation; rusticity, rapid growth, and fruits dispersed by birds are desirable attributes for planting in ecosystem restoration^2^. At the same time, the beauty of its foliage and its fructification make it used worldwide for urban landscaping and planting in streets, parks, and gardens^1^. Also, its fruits are used as a condiment in cooking (pink peppercorns or “poivre rose”), its flowers are honey-bearing, and different parts of the plant have medicinal uses^1,3^. These attributes justify the international interest in species cultivation.

Seedling commercial production requires quality substrates to ensure better conditions for plant growth. A suitable substrate can physically supportthe seedling growth, allowing the exchange of gases and guaranteeing the water and nutrient supply to the plants^4^. The substrate choice must consider chemical (absence of toxic substances, nutrient content, and others), physical (porosity, density, and others), biological (absence of pathogens), and economic factors (local availability and costs)^4,5^. Many commercial substrates (CS) for tree species seedlings are available in the market, but nurseries cannot always acquire these products locally at affordable prices^6^. This reality makes producers prepare their substrate using locally available materials, often urban or agro-industrial solid waste.

Solid waste from sewage treatment is known as sewage sludge (SS) and has been tested and approved as a substrate for seedling production of forest species^7,8^. This material is being used in the Rio de Janeiro State Water and Sewage Company (CEDAE) nurseries, which produced more than 1,000,000 seedlings between 2015 and 2019^9^. SS is rich in organic matter and nutrients; it can be considered suitable for substrate, organic compost, or soil conditioner after undergoing stabilization, drying, and composting^10^. In addition, SS is widely available in urban centers worldwide; it can be used as a substrate to grow container seedlings for different purposes (street trees, forest restoration, silviculture, and others) in different regions or countries. Using SS as a substrate can be considered more sustainable than disposal in landfills, which is the most common practice in Brazil^11,12^.

Proper application of fertilizers is one of the main factors responsible for productivity and quality in forest seedlings production^13,14^. Controlled-release fertilizers (CRF) are the most commonly used fertilizers in forest nurseries. They are coated with a layer of water-permeable organic resin^5,14^, which controls the release of nutrients, synchronizing the nutritional demand of the seedlings with the availability of nutrients and reducing losses through leaching^5^.

The substrate is the nursery input that is more associated with the availability of nutrients for seedlings and the need for fertilization^14^. In forest nurseries that use polypropylene tubes as containers, the substrates tend to have low levels of nutrients, such as commercial formulations with sphagnum peat or pine bark^6^. The main concern is that the substrate has ideal physical characteristics since chemical fertilizers can correct or prevent nutritional deficiencies^15^. According to Ribeiro et al. (2009)^16^, the fertilizer application can be reduced by up to 50% when using SS as a substrate. Considering the chemical fertilizers crisis and the increase in prices the world is experiencing, alternative materials for plant nutrition should be researched. Therefore, the joint study of these two factors (substrate and fertilizer rates) allows evaluating and recommending of different fertilizer rates according to the characteristics of each substrate.

The use of SS in the substrate for producing *Schinus terebinthifolia* seedlings can be considered viable or even beneficial, having been evaluated by Nóbrega et al. (2007)^17^, Trigueiro and Guerrini (2014)^18^, Abreu et al. (2018)^19^, among other authors. In addition, other studies have evaluated substrates containing SS compared to CSs^20–22^ or the effects of chemical fertilizers^7,16,23^ in forest seedlings production. However, the evaluation of different substrates combined with CRF rates is little studied for Atlantic Forest species, especially when considering a substrate containing 100% SS, as evaluated in the present study.

Accordingly, the study’s hypotheses were: *Schinus terebinthifolia* seedlings could be produced in a substrate containing 100% of stabilized SS without adverse effects of the SS’s physical and chemical characteristics on the seedlings; the seedlings produced with SS can achieve similar growth and quality to those produced with the CS; considering the nutrient content of the SS, it would be possible to produce seedlings without chemical fertilizers.

Similar studies generally evaluate only one factor (substrate or fertilization), fewer fertilizer rates (which affects the regression analysis adjustment), and the use of SS in variable proportions as a substrate component. The present study assessed a factorial experiment (2 substrates × 4 CRF rates), substrates composed of 100% SS, and if it is possible to produce seedlings using only SS as substrate, which can impact seedlings’ production costs avoiding costs with CS and CRF.

Thus, the objective of this work was to evaluate the effects of two substrates (100% SS from Ilha WWTP and CS made of organic materials (mainly sphagnum peat)) under four different CRF rates (0, 3, 6, and 12 kg m^−3^) on the growth and quality of *Schinus terebinthifolia* Raddi (rose pepper) seedlings.

## Material and methods

### Experiment description

The experiment was carried out in the forest nursery of the “Companhia Estadual de Águas e Esgotos do Rio de Janeiro” (CEDAE), located at the Guandu Water Treatment Plant, municipality of Nova Iguaçu, state of Rio de Janeiro, Brazil, between August and December 2019, totaling 110 days. The local climate is Aw according to the Köppen-Geiger classification, which means tropical with dry winters and rainy summers.The experimental design was completely randomized with a factorial 2 × 4 scheme (substrates × rates), consisting of two substrates (SS from Ilha WWTP and a CS made of mainly sphagnum peat) and four CRF rates (0, 3, 6, and 12 kg m^−3^).

The SS was provided by CEDAE, coming from the wastewater treatment plant (WWTP) of Ilha do Governador, Rio de Janeiro, RJ, Brazil. The Ilha WWTP performs sludge treatment at the secondary level using the activated sludge system. The secondary sludge removed from the decanters is condensed in a centrifuge and sent to stabilize in an anaerobic digester. Then the material is sent to full-sun drying beds, where it remains for at least 90 days, reaching humidity below 30%. According to CEDAE, this SS batch showed values for pathogenic microorganisms and heavy metals lower than those allowed by CONAMA Resolution nº498/2020, with *Escherichia coli* of11.39 MPN g-1 TS (most probable number per gram of total solids) absent for *Salmonella* sp. And for heavy metals (in mg kg^−1^): As < 0.011; Ba 223.6; Cd 1.5; Pb 95.8; Cu 247.2; 60.2 Cr; Ni 33.6 Se < 0.011; Zn 1008.7. The CS was purchased locally from a widely used brand in forest nurseries. The main component of this substrate is sphagnum peat (70%), vermiculite (30%), and slight base fertilization, resulting in electrical conductivity of 0.70 dS m^−1^. The substrates were subjected to chemical, physical–chemical, and physical analysis before applying fertilizer rates (Table 1).

**Table 1:** Total and available contents (in parentheses) of nutrients, electrical conductivity (EC), C/N ratio, and organic matter (OM) of the substrates used for producing *Schinus terebinthifolia* seedlings.

Parameters	Substrates	
	SS	CS
N total (g kg^−1^)	18.3	5.8
P total/available (g kg^−1^)	7.6/0.16	2.8/0.11
K total/available (g kg^−1^)	1.3/0.16	2.5/0.50
Ca total/available (g kg^−1^)	12.8/2.54	15.8/2.08
Mg total/available (g kg^−1^)	1.9/0.15	26.1/1.48
Ph	5.7	5.8
Electrical conductivity (dS m^−1^)	2.65	0.72
Organic matter (%)	33.7	52.4
C/N	8.2	40.8
Density (g cm^−3^)	0.55	0.17
Total porosity (%)	75.5	79.7
Macroporosity (%)	34.3	35.3
Microporosity (%)	41.2	44.4

In which: *CS* commercial substrate based on sphagnum peat; *SS* substrate composed 100% of sewage sludge from the Ilha do Governador WWTP. Total contents determined by ICP-OES; C and N by CHN-600; available P and K levels with Mehlich 1 extractor, Ca and Mg per KCl  − 1 mol l^−1^; OM by multiplying the C content by the “Van Bemmelen” factor, pH in pH-meter; and EC in conductivity meter. Density and porosity were determined by weighing soaked, drained, and dried samples in a cylinder of known volume.

Four rates of controlled-release fertilizer (CRF) NPK (15–09-12) were applied (0, 3, 6, and 12 kg m^−3^ of substrate). This fertilizer also contains 1% Mg, 2.3% S, 0.05% Cu, 0.45% Fe, 0.06% Mn, and 0.02% Mo in its composition. Top-dressing fertilization was not performed as the CRF gradually releases nutrients to plants, and Schinus terebinthifolia is a fast-growing species. Thus, considering the amplitude between the tested CRF rates, it was supposed that the seedlings would have their nutritional demands fulfilled at some of the tested rates.

Each substrate was placed in polypropylene tubes of 280 cm^3^. The tubes were filled manually with the substrate of each treatment. Three seeds were sown per container. The seedlings were grown in a full-sun nursery area throughout the production period. Thinning was performed 20 days after sowing, leaving the larger and more centralized seedling. The seedlings were irrigated two or three times a day at the beginning and (eventually) end of the morning and late afternoon for ten minutes for each irrigation to maintain 60 to 70% of the substrate’s field capacity. The seedlings were alternated at 50 days after sowing for a density of 50% of the cells in the tray, which was maintained until the end of the experiment.

### Experiment evaluation

At 110 days after sowing, seedlings were measured, considering the plants from most treatments reached the proper size for planting in forest restoration (height of 20–40 cm and diameter greater than 3 mm, according to Souza Junior and Brancalion, 2016^24^). The shoot height was measured with a graduated ruler, and the stem diameter with a digital caliper. Six seedlings per sample were randomly selected to evaluate shoot (SDM) and root dry mass (RDM). In this process, the seedlings’ shoot and the root system were separated and dried in a forced-air circulation oven at 65 ºC for 72 h. With these data, the parameters height/diameter ratio (H/D), shoot/root ratio (S/R), and Dickson’s quality index (DQI) was calculated using the formula below.

$${\text{Dickson}}{\prime }{\text{s~}}\;{\text{quality~}}\;{\text{index}}\;{\text{~(DQI)~ = ~}}\frac{{{\text{TDM}}}}{{\left[ {\left( {\frac{{\text{H}}}{{\text{D}}}} \right){\text{ + ~}}\left( {\frac{{{\text{SDM}}}}{{{\text{RDM}}}}} \right)} \right]}}$$

In which: TDM—total dry mass; H—height; D—diameter; SDM—shoot dry mass; and RDM—root dry mass.

### Statistical analyses

Statistical analyzes and graphs were generated using R statistical software^25^. Initially, the data was submitted to a descriptive statistic, illustrated by boxplot graphs with the package “ggpubr” (Kassambara, 2020^26^). In the sequence, a linear correlation analysis was performed between the variables with the Spearman coefficient since some variables did not present a normal distribution according to the Shapiro–Wilk test. The correlation analysis was performed using functions of the R base, and the results were illustrated with the package “corrplot” (Wei and Simko, 2021^27^). The plots illustrating these analyses are presented in the Supplementary Information (see Supplementary Fig. S1, S2, S3, S4, and S5).

Multivariate analyses were performed to evaluate seedlings’ quality considering the morphological variables H, D, H/D, SDM, RDM, S/R, and IQD. The treatments were used as a reference to evaluate the observations’ grouping in the PCA’s biplot. The data adequacy for multivariate analyses was tested by the KMO index and the Bartlett Test of Sphericity using functions of the package “psych” (Revelle, 2021^28^). Since the KMO index presented a value of 0.60, it was decided to perform the principal components analysis (PCA) to evaluate the grouping of observations and the variables. A k-means cluster analysis was also performed to evaluate the observations’ grouping. The cluster analysis results were plotted in a PCA biplot. The multivariate analyses were performed using the base R functions (package “stats”), and the package “factoextra” (Kassambara and Mundt, 2020^29^) was used to plot the results. It is presented in Supplementary Information (Fig. S6, S7, S8, S9, and S11) some additional plots that supported the identification of the ideal number of principal components, clusters, and/or helped us interpret the results.

Experimental data statistics were performed using the “ExpDes.pt” package^30^. The data were submitted for analysis of variance, followed by F-test evaluating the interaction between the factors for each variable. Tukey’s test was used to compare the means of the substrate factor within the CRF rates factor. CRF rates within each substrate were subjected to regression analysis. The residuals normality was evaluated by the Shapiro–Wilk test and the homogeneity of the variances by the Bartlett test. The data were transformed whenever needed. After the transformation, it was verified again if the assumptions were met. The graphs of the regression curves were prepared using the “ggplot2” package^31^.

## Results

The interaction between the two factors (substrates and CRF rates) was statistically significant by the F-test (*p* < 0.05) for all variables (Table 2). Thus, the results were evaluated by simple effects tests, meaning that the levels (treatments) of one factor were tested inside the levels of the other factor.

**Table 2:** Analysis of variance (ANOVA) of the variables measured in *Schinus terebinthifolia* Raddi seedlings at 110 days after sowing, considering the effect of the substrates in each of the controlled-release fertilizer (CRF) rates.

Variables	Response	Df	Sum Sq	Mean Sq	F Value	Pr (> F)
log(Height)	Substrate	1	2.59	2.59	534.86	≈ 0.00
	CRF rates	3	8.69	2.90	599.52	≈ 0.00
	Interaction	3	7.70	2.57	531.03	≈ 0.00
	Residuals	24	0.12	0.005		
log(Diameter)	Substrate	1	1.37	1.37	581.71	≈ 0.00
	CRF rates	3	4.37	1.46	620.67	≈ 0.00
	Interaction	3	4.41	1.47	625.92	≈ 0.00
	Residuals	24	0.06	0.002		
H/D	Substrate	1	5.41	5.41	31.65	≈ 0.00
	CRF rates	3	32.16	10.72	62.77	≈ 0.00
	Interaction	3	24.24	8.08	47.31	≈ 0.00
	Residuals	24	4.01	0.17		
SDM	Substrate	1	8.09	8.09	13.94	0.0010
	CRF rates	3	12.73	4.24	7.31	0.0011
	Interaction	3	14.79	4.93	8.50	0.0005
	Residuals	24	13.92	0.58		
RDM^−2.7^	Substrate	1	0.0011	0.0011	63.86	≈ 0.00
	CRF rates	3	0.0018	0.0006	34.76	≈ 0.00
	Interaction	3	0.0017	0.0005	31.24	≈ 0.00
	Residuals	24	0.0004	0.00001		
S/R	Substrate	1	0.0004	0.0004	0.03	0.86
	CRF rates	3	0.0622	0.0207	1.55	0.23
	Interaction	3	0.6812	0.2271	17.01	≈ 0.00
	Residuals	24	0.3204	0.0133		
DQI	Substrate	1	0.05	0.05	1.53	0.228
	CRF rates	3	0.14	0.05	1.28	0.304
	Interaction	3	0.60	0.20	5.56	0.004
	Residuals	24	0.87	0.04		

In which: *CRF* controlled-release fertilizer; *DQI* Dickson’s quality index; *H/D* height/diameter ratio; *SDM* shoot dry mass; *RDM* root dry mass; and *S/R* shoot/root ratio.

### Substrates inside controlled-release fertilizer rates

The results for the substrate factor inside the CRF rates (Table 3) show that the SS presented the highest value for all variables at the zero rate, except for DQI, where the treatments did not differ. Fertilization with 3 kg m^−3^ equaled the values ​​between the substrates for diameter, shoot, and root biomass, with higher height and H/D in the SS and better DQI in the CS. The difference between the substrates at the 6 kg m^−3^ was smaller, with the SS showing the highest value for RDM and the CS the highest value for S/R. Higher values were observed ​​for the CS in height, H/D, and S/R at the highest rate, while higher values were found ​​for the SS in root biomass and DQI and similar values ​​for diameter and shoot biomass.

**Table 3:** Means and standard deviation (in parentheses) of the variables measured in *Schinus terebinthifolia* Raddi seedlings at 110 days after sowing, considering the effect of the substrates in each of the controlled-release fertilizer (CRF) rates.

Variables	Substrates	CRF rates (kg m^−3^)			
		0	3	6	12
Height (cm)	SS	37.4a (4.0)	40.8a (1.4)	44.1a (1.9)	36.0b (2.1)
	CS	4.0b (0.3)	31.6b (3.6)	45.1a (1.4)	44.1a (1.0)
Diameter (mm)	SS	5.2a (0.2)	5.1a (0.3)	5.5a (0.3)	5.0a (0.2)
	CS	0.9b (0.1)	5.3a (0.4)	5.2a (0.2)	5.3a (0.3)
H/D	SS	7.2a (0.7)	7.9a (0.2)	8.0a (0.2)	7.2b (0.2)
	CS	4.1b (0.4)	5.9b (0.4)	8.6a (0.2)	8.3a (0.6)
SDM (g)	SS	6.4a (0.9)	6.5a (0.7)	6.2a (0.6)	6.3a (1.2)
	CS	3.1b (0.1)	5.8a (0.8)	6.1a (0.5)	6.4a (0.9)
RDM (g)	SS	4.9a (0.3)	4.5a (0.2)	5.7a (0.7)	5.8a (1.1)
	CS	3.0b (0.1)	5.1a (0.9)	4.5b (0.3)	4.6b (0.6)
S/R	SS	1.3a (0.1)	1.4a (0.1)	1.1b (0.1)	1.1b (0.2)
	CS	1.0b (0.0)	1.1b (0.1)	1.3a (0.0)	1.4a (0.1)
DQI	SS	1.3a (0.2)	1.2b (0.1)	1.3a (0.1)	1.5a (0.3)
	CS	1.2a (0.1)	1.5a (0.3)	1.1a (0.1)	1.1a (0.2)

In which: *CS* commercial substrate; *SS* sewage sludge; *DQI* Dickson’s quality index; *H/D* height/diameter ratio; *SDM* shoot dry mass; *RDM* root dry mass; and *S/R* shoot/root ratio. NOTE: means followed by the same letter on the same column and for the same variable do not differ by Tukey’s test at the 5% significance level.

The SS showed the highest means regarding RDM for all rates, except for 3 kg m^−3^, in which the substrates did not differ (Table 3). The results for S/R were higher for the SS at low rates (0 and 3 kg m^−3^) and higher for the CS at high rates (6 and 12 kg m^−3^). The DQI presented relatively close values (even when a significant difference was observed) between the treatments.

### Controlled-release fertilizer rates inside the substrates

**Figure 1:**
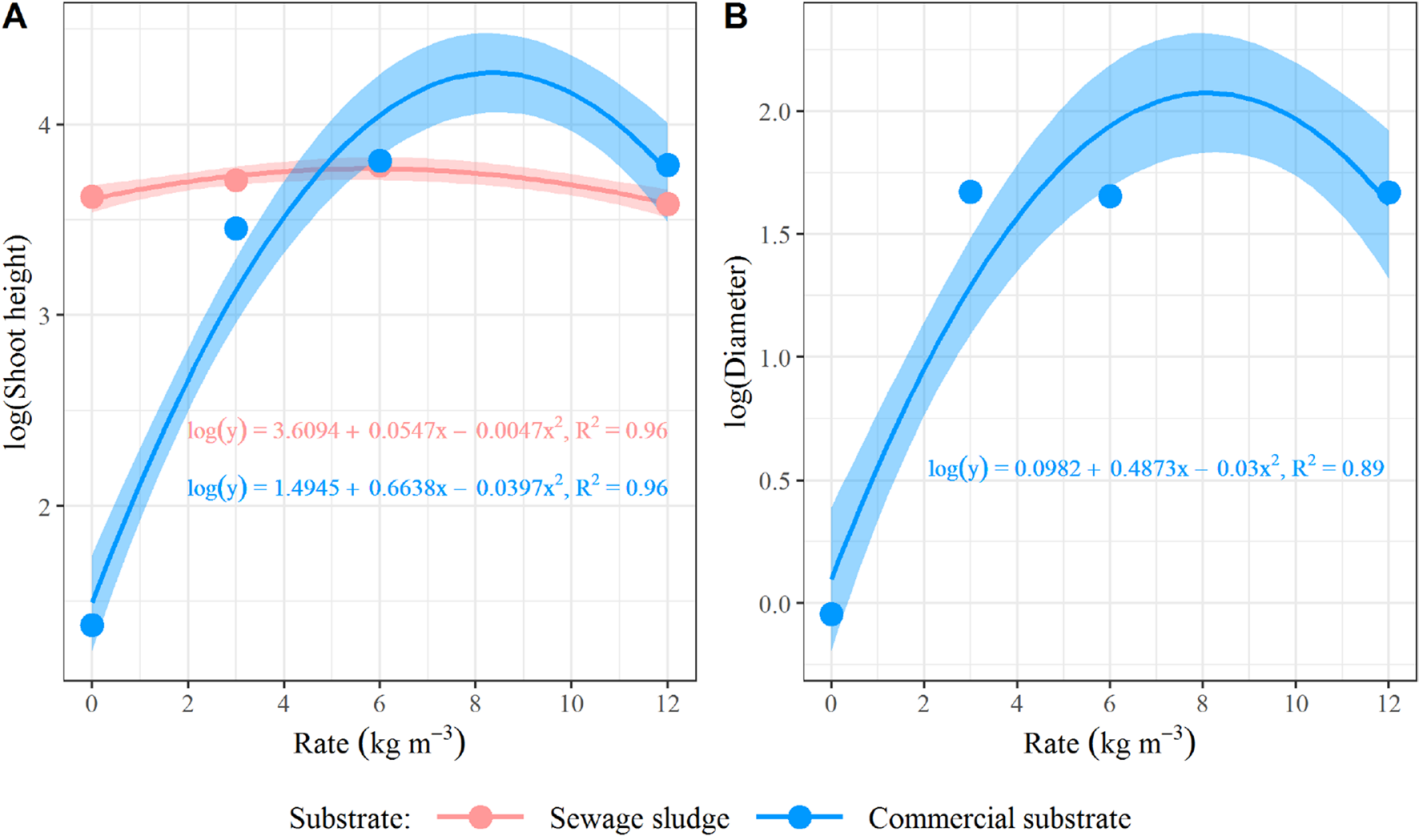
Curves for (**A**) growth in height and (**B**) in diameter of *Schinus terebinthifolia* Raddi seedlings at 110 days after sowing, considering the effect of controlled-release fertilizer rates on the evaluated substrates. PS.: regression models were considered significant at the 5% level; the dots represent the mean value of samples in each observation, and the shade represents the 95% confidence interval.

Considering the effects of the CRF rates factor on the substrate factor, it was observed significance (p < 0.05) for height in both substrates. In contrast, for diameter, the rates were only significant in the CS (Fig. 1). The mean value of the different CRF rates in the SS was relatively close for diameter and height (even when differences between rates were observed), indicating that the response of seedlings to fertilizer application was low.

**Figure 2:**
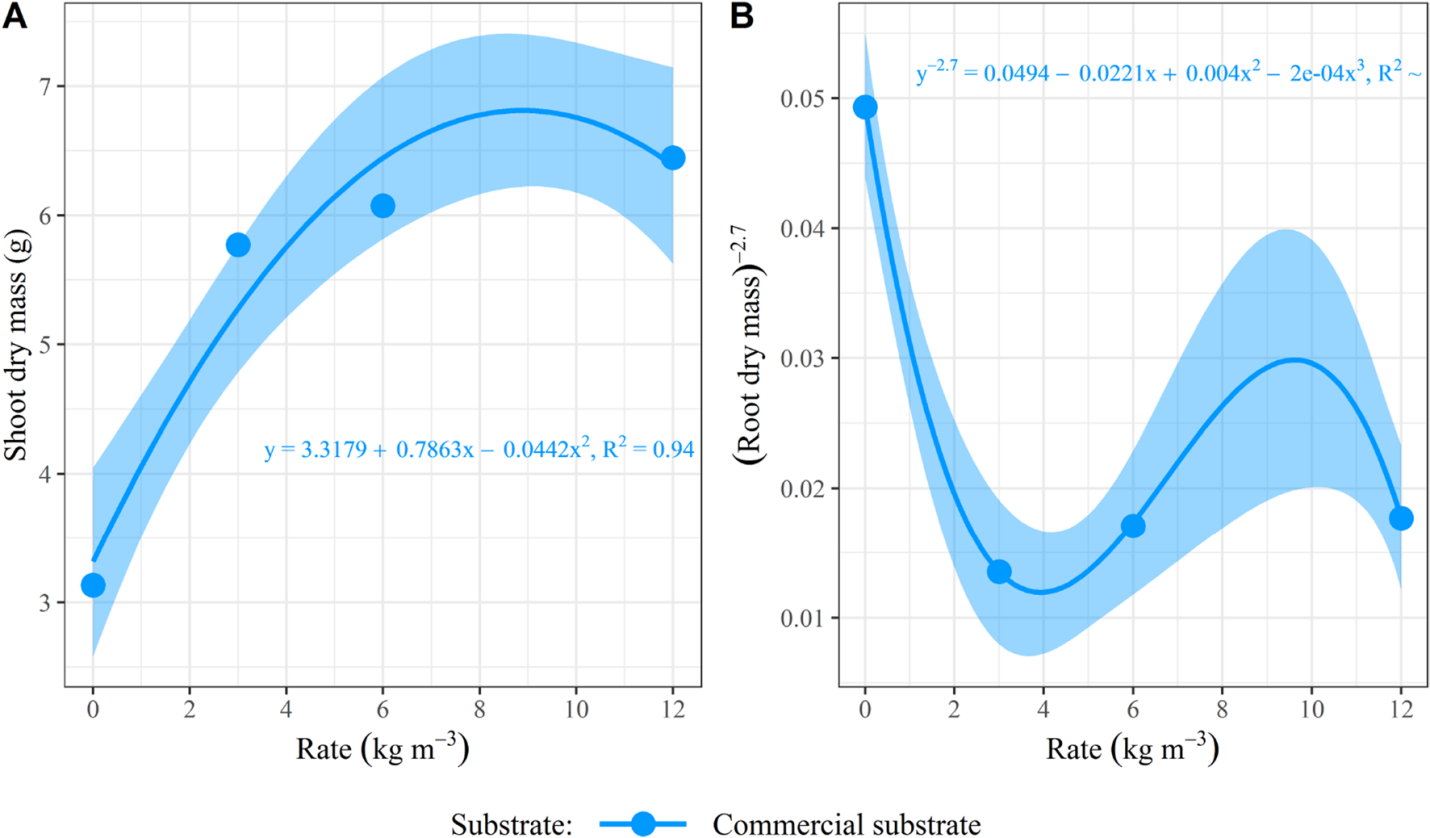
Curves for (**A**) shoot and (**B**) root dry mass in *Schinus terebinthifolia* Raddi seedlings at 110 days after sowing, considering the effect of controlled-release fertilizer rates on the evaluated substrates. PS.: regression models were considered significant at the 5% level; the dots represent the mean value of samples in each observation, and the shade represents the 95% confidence interval.

It is observed that the seedlings in the CS responded to the CRF rates in a quadratic way for SDM and in a cubic way for RDM (Fig. 2). As only four CRF rates (levels) were tested, an overfit occurs when considering the cubic model, as in the cases of RDM (Fig. 2B) and the DQI (Fig. 4). The CRF rates were constant in the SS for biomass (SDM and RDM).

**Figure 3:**
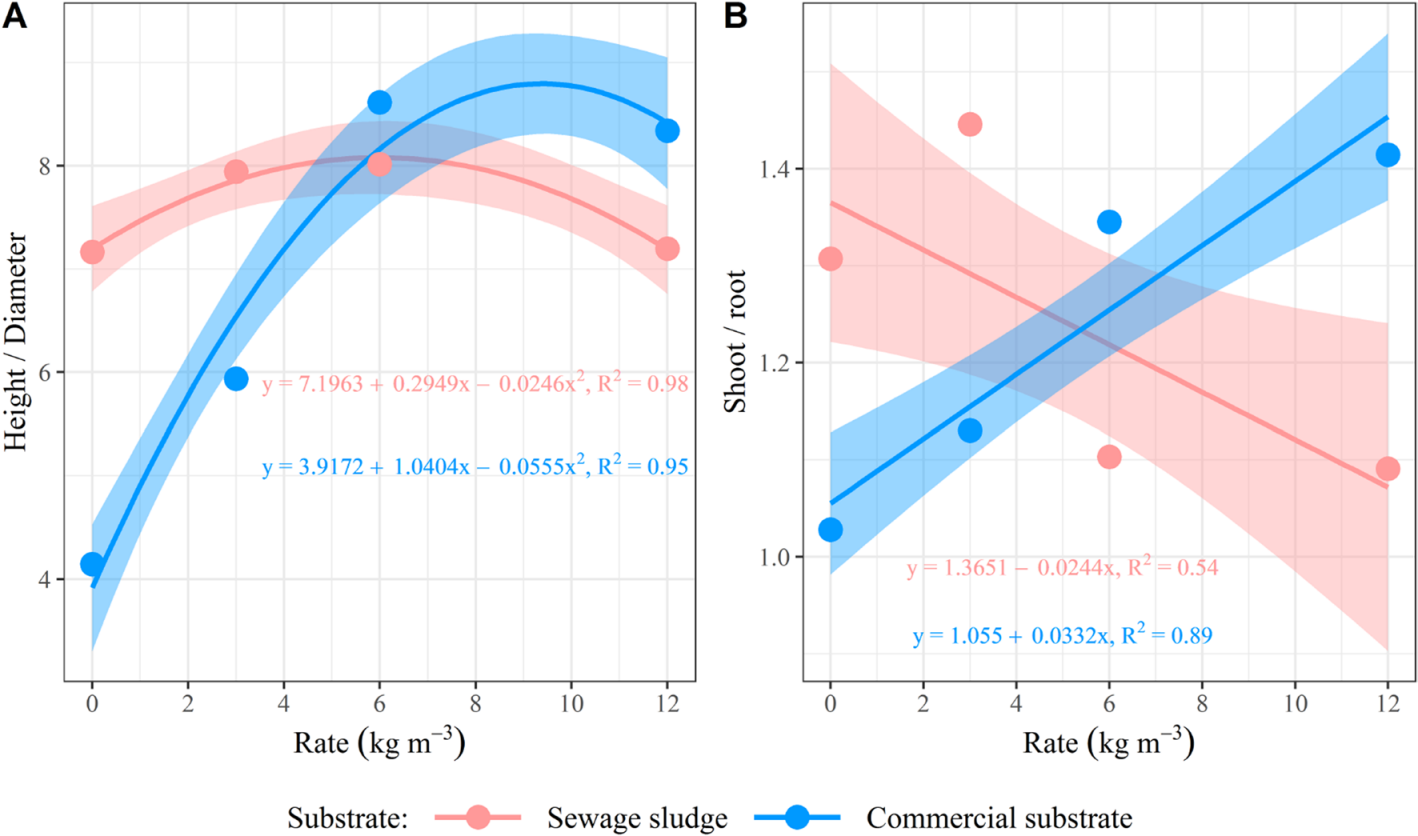
Curves for (**A**) height/diameter ratio; and (**B**) shoot/roots ratio in *Schinus terebinthifolia* Raddi seedlings at 110 days after sowing, considering the effect of the controlled-release fertilizer rates on the evaluated substrates. PS.: regression models were considered significant at the 5% level; the dots represent the mean value of samples in each observation, and the shade represents the 95% confidence interval.

**Figure 4:**
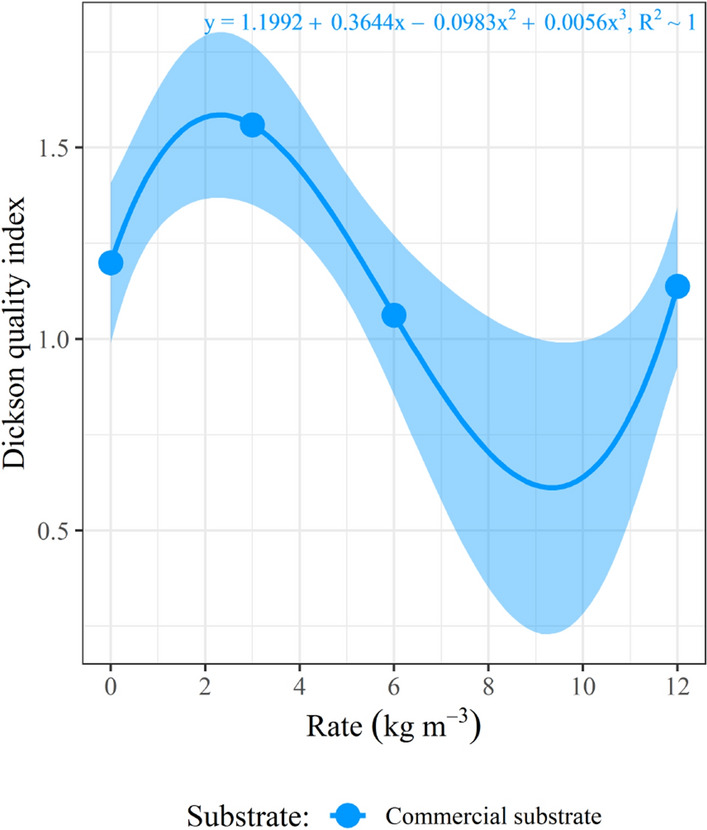
Curves for Dickson quality index in *Schinus terebinthifolia* Raddi seedlings at 110 days after sowing, considering the effect of the controlled-release fertilizer rates on the evaluated substrates. PS.: regression models were considered significant at the 5% level; the dots represent the mean value of samples in each observation, and the shade represents the 95% confidence interval.

A quadratic effect for H/D, linear for S/R (Fig. 3), and cubic for DQI (Fig. 4) was observed for the CS under growing CRF rates. There was no effect of the CRF rate for the SS on the DQI, the H/D presented a quadratic pattern, and S/R was linear, being reduced as the rates were increased.

### Recommended fertilizer rates

It is observed that an average addition of 7.8 kg m^−3^ of CRF would be necessary for the seedlings to present their maximum growth in the CS (Table 4). The rates for the SS were only significant for height, requiring the application of 5.8 kg m^−3^ of CRF to achieve maximum growth. It is worth mentioning that the variables referring to the quality of the seedlings were not included in this evaluation since higher values​​ in these do not always indicate higher quality. The RDM was also not considered in this calculation since the rates were not significant for the SS and fitted a cubic model for the CS.

**Table 4:** Maximum values for the height, diameter, and shoot dry mass (in parenthesis, the standard error) and the respective CRF rates to reach them in producing *Schinus terebinthifolia* Raddi seedlings at 110 days after sowing produced in two substrates and different controlled-release fertilizer rates.

Parameters	Sewage sludge		Commercial substrate		Difference between substrates*
	Rate (kg m^−3^)	Maximum	Rate (kg m^−3^)	Maximum	(kg m^−3^)
Height (cm)	5.8	43.3 (1.08)	8.4	71.5 (2.77)	4.3
Diameter (mm)	n.s	n.s	8.1	8.0 (2.05)	4.3
SDM (g)	n.s	n.s	6.8	8.9 (1.31)	5.8
Mean rate	5.8	–	7.8	–	4.8

*This value is equivalent to the fertilizer rate to be added to the commercial substrate so that it reaches the values of each variable observed for the sewage sludge substrate without fertilizer addition. The values were estimated considering a 5% significance level.

### Multivariate analyses

**Figure 5:**
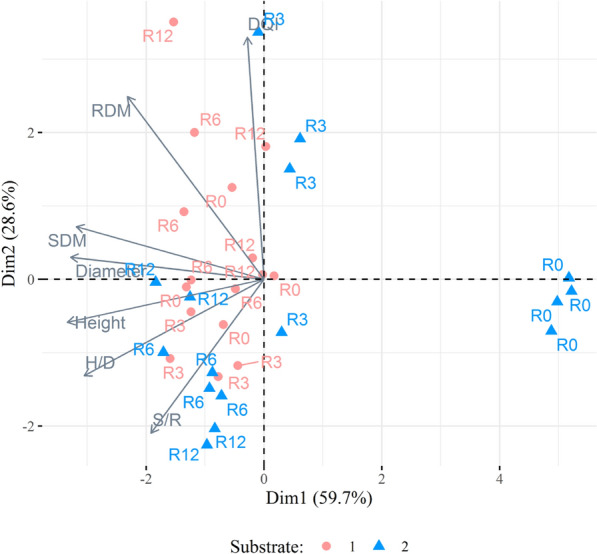
Principal component analysis of *Schinus terebinthifolia* Raddi seedlings at 110 days after sowing produced in two substrates and four controlled-release fertilizer rates (R0, R3, R6, and R12). In which: DQI: Dickson’s quality index; H/D: height/diameter ratio; SDM: shoot dry mass; RDM: root dry mass; and S/R: shoot/root ratio.

The PCA represented 88.3% of the data variance in the two dimensions presented in Fig. 5, demonstrating the method’s capacity to explain the data. The samples and variables were grouped into three groups. Regarding the variables, the Height, Diameter, H/D, and SDM were grouped between the lower and upper left quadrant with a high negative correlation with PC1 and weak correlation with PC2 (positive for diameter and SDM; negative for height and H/D). The Dickson quality index (DQI) and RDM were grouped in the upper left quadrant with a high positive correlation with PC2. And the last group was composed only by the S/R with moderate to strong negative correlation with both PC1 and PC2. These results for variables grouping were confirmed by hierarchical cluster (Supplementary Fig. S11).

**Figure 6:**
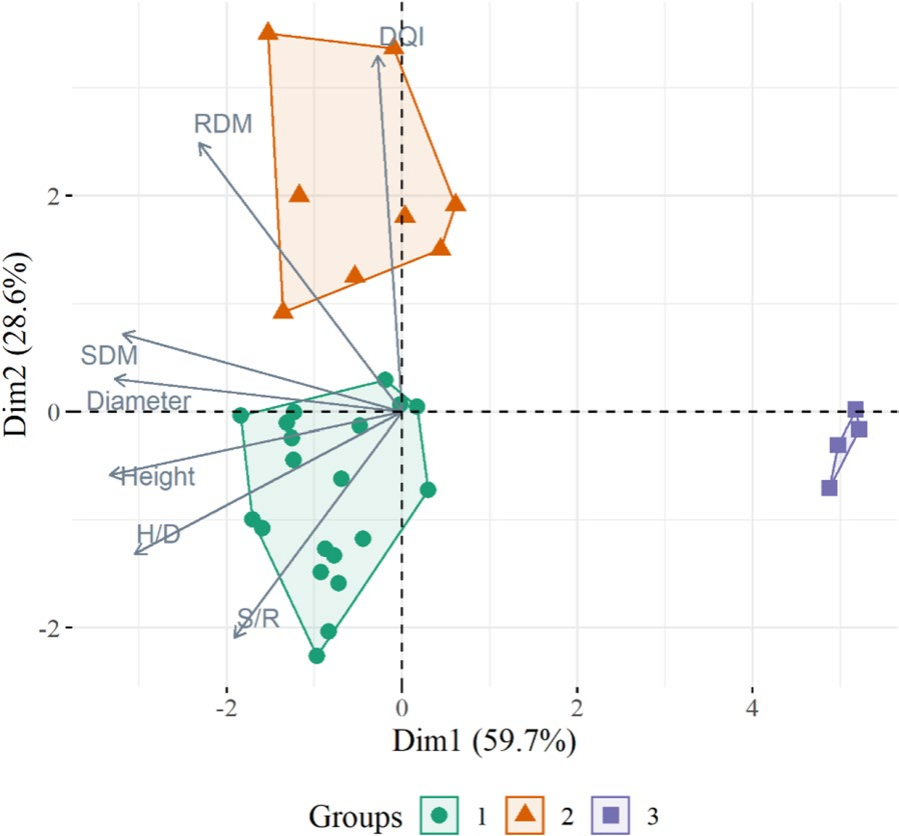
Principal component analysis considering the samples’ grouping by cluster analysis of *Schinus terebinthifolia* Raddi seedlings at 110 days after sowing produced in two substrates and four controlled-release fertilizer rates. In which: DQI: Dickson’s quality index; H/D: height/diameter ratio; SDM: shoot dry mass; RDM: root dry mass; and S/R: shoot/root ratio.

The first group of samples is located from the center of the graph to the lower left quadrant, formed by a similar number of samples from the two substrates. It is presented starting from samples with higher ​​S/R, H/D, height, and diameter to samples with average values ​​in these and other variables (Figs. 5 and 6). The second group is in the upper left quadrant, mainly formed by SS samples, and has higher DQI and RDM. The third group is in the center and to the right; it is formed by the four samples of CS that did not receive fertilizers and presented low values ​​for the growth and quality variables.

Samples were grouped in three clusters by the cluster analysis as verified in Fig. 6, where the clusters are plotted inside the PCA’s biplot. It is possible to observe that both PCA and cluster analyses resulted in a similar grouping of the samples. Group 1 contains seedlings with adequate quality, as these samples are correlated with the main morphological growth and quality parameters. Group 2 includes samples more correlated with higher root mass and DQI. While group 3 contains the seedlings from CS with no fertilization, which showed inferior growth and quality. The mean values of the morphological parameters for the observations in each cluster confirmed these results (Supplementary Table S10).

#### Discussion

### Substrates inside controlled-release fertilizer rates

The chemical properties of each substrate explain the difference observed between them when CRF was not applied (0 kg m^−3^) since the seedlings did not grow in the CS and showed satisfactory growth in the SS (Table 1). Despite receiving base fertilization in its formulation, the CS presented low total nutrient contents, and the available values tend to be leached after the first weeks of daily irrigation. In contrast, the SS has considerable total nutrient contents and less available values, illustrating that nutrients in this substrate are gradually released to the plants, similar to a CRF application^32^. Therefore, when seedlings are produced with the CS, there is a need for top-dressing fertilization, base application of CRF rates, or similar input to supply the demanded nutrients for proper seedling growth^6,13^.

With growing CRF rates, the seedling growth in the CS was equal to or even exceeded that observed for the SS. Similar results were also observed by Trigueiro and Guerrini (2014) for *Schinus terebinthifolia*^18^. When using base fertilization and fertirrigation, the authors observed that a CS based on pine bark produced seedlings with better growth and quality than a substrate containing 80% SS and 20% carbonized rice husk^18^. These results were attributed to the superior physical characteristics of the CS^18^.

The ideal physical characteristics of a substrate for seedling production in polypropylene tubes are low density (0.25 to 0.40 g cm^−3^), high total porosity (75 to 85%), and macroporosity (35 to 45%)^6,33^. Hence, CS can be regarded as superior to SS, considering the physical properties (Table 1). Thus, it is possible to affirm that the CRF application compensated for the deficiency in the CS’s chemical properties, promoting conditions that potentiated the growth of the seedlings. Adding SS to the substrate generally increases density, microporosity, and water retention capacity. On the other hand, it decreases macroporosity, aeration, and drainage capacity^15,16^. Therefore, considering the physical and chemical properties of the SS, the CRF application did not substantially affect the growth of seedlings in this substrate.

Regarding quality, plants in high nutrient availability tend to have higher S/R and prioritize shoot growth, specifically leaves, to increase their photosynthetic capacity^34,35^, as occurred in the CS with high CRF rates. However, higher rates promoted more significant root growth and lower S/R for SS, which can be attributed to a characteristic of the species. Some studies reported higher *Schinus terebinthifolia* RDM on substrates with higher proportions of SS^17,18^, while the opposite was verified by others^19,36,37^.

High ​​H/D values indicate tall and thin seedlings, also known as etiolated, with inferior quality since they are less resistant to handling, the action of winds, frosts, and droughts^38^. Values ​​below 10 for pioneer and fast-growing species such as *Schinus terebinthifolia* can be considered adequate^19,38^. Therefore, it is possible to affirm that all treatments presented acceptable values ​​for this parameter.

For the DQI, the reference values for the species are highly variable^33,38^. Abreu et al. (2018) observed that *Schinus terebinthifolia* seedlings with DQI between 0.41 and 1.49 showed 100% survival 12 months after outplanting^19^. When using these values as a reference, it can be concluded that all treatments evaluated in the present study produced quality seedlings—demonstrating how inefficient it is to use only one parameter to assess the seedlings’ quality^33,39^. The seedlings produced in the CS without CRF, despite the high DQI indicating balanced growth, showed much lower height and diameter than seedlings considered of good quality for this species^24^.

### Controlled-release fertilizer rates inside the substrates

In SS and CS, the height presented a quadratic response, and for the diameter, the rates were significant only for the CS, which showed a quadratic pattern (Fig. 1). Height and diameter can be considered the main morphological parameters used in evaluating forest seedlings, as they can be measured easily, with low costs, and are non-destructive^33,38,39^. Except for the treatment with the CS at a rate of 0 kg m^−3^, all the others produced *Schinus terebinthifolia* seedlings suitable for planting. According to Gonçalves et al. (2000), the recommended height is between 20 and 35 cm and diameter between 5 and 10 mm for Atlantic Forest species^40^. When considering the recommendation by Souza Junior and Brancalion (2016)^24^, specific to the *Schinus terebinthifolia*, the seedlings are suitable for planting with a height of 20-40 cm, a diameter greater than 3 mm, and a production time of 3 to 4 months (90 to 120 days).

Thus, considering an average height above 30 cm and a diameter above 3 mm, it is observed that the seedlings produced in this study with SS at any rate and CS with CRF rates equal to or greater than 6 kg m^−3^ could be ready for planting in less than 110 days. Cabreira et al. (2017) observed that CRF application accelerated the growth of *Schinus terebinthifolia* seedlings, allowing them to be produced in a shorter time, reaching the recommended height in 90 days after transplanting^23^. As more extended time in the nursery implies higher expenses with maintenance, irrigation, and productive area occupation^41^, reducing production time may be advantageous to justify higher CRF rates.

The lack of response to CRF rates in the SS for biomass accumulation reinforces that no benefit was obtained from fertilizer application for seedlings produced in a substrate with 100% SS. Since the seedlings showed adequate shoot and root biomass in the SS regardless of the CRF rate, they can be produced in this substrate without fertilizers.

For RDM in the CS, the adjusted cubic model suggested a condition not observed in measured values, where the highest values ​​would be reached without CRF application (Fig. 2B). In addition, another growth peak can be estimated at a rate higher than 12 kg m^−3^, while the other variables suggest a depression of values ​​at this rate. This occurred because the data was transformed to meet the assumption of homogeneity of variances. The “box cox” transformation suggested a negative value, which resulted in an inversion in the direction of the curve.

The seedlings in the CS showed a cubic response pattern to the CRF rates for the variables RDM and DQI (Figs. 3B and 4). As only four CRF rates (levels) were tested, overfitting occurs when considering the cubic model in the present study. Therefore, the R^2^ for this model has a value of approximately 1 (one), and the curve always passes over the midpoints, suggesting a perfect adjustment of the model. It is recommended that future experiments studying fertilizer rates in seedling production should consider testing five or more fertilizer rates to avoid overfitting regression models.

A quadratic model, such as the one fitted for height, diameter, and SDM in the CS, would be more suitable for RDM and DQI, considering the “law of diminishing returns.” This concept mentions that the most significant increase in production is obtained in the first fertilizer rate applied; the increments then tend to get smaller with successive applications of equal amounts of the same fertilizer, reaching a point of stagnation or even depression in higher rates^42^.

In the present study, the increasing CRF rates promoted greater S/R in the CS (Fig. 3B). As already stated, seedlings in a high fertility environment tend to invest in producing leaves to increase photosynthesis^35^. Meanwhile, an inverse trend was observed for the SS, in which increasing rates led to a decrease in S/R. The shoot root ratio (S/R) represents the biomass distribution and is related to the seedlings’ water balance^39^, acceptable values ​​for this parameter are usually between 1 and 3^38^. Low S/R values indicate deficient leaf development and lower photosynthesis potential^43^. In contrast, high ​​ S/R values may suggest that seedlings are more vulnerable to water stress since their transpiration surface (shoot) is more expressive than their water absorption potential (root)^44^.

Seedlings produced with SS as substrate responded to CRF rates only for height in the present study, which can be considered unusual. The variation between the SS batches can explain the differences observed between the present study and others that tested SS as a substrate for tree seedlings^8,23,45^. The SS's physical, chemical, and biological characteristics are decisive in indicating its use as a substrate or not. It is necessary to observe factors such as high density, low porosity, salinity, pH, pathogenic microorganisms, and potentially toxic substances^16,20,43,46^. These and other factors vary between SS from different WWTPs and even in different batches from the same plant^10^.

Evaluating the production of *Schinus terebinthifolia* seedlings, Bonnet et al. (2002) observed that thermally dried SS could be applied in proportions of up to 15% of the substrate due to its high pH^46^. While for the composted sludge, the authors found it was possible to produce seedlings in a substrate with 100% SS, although proportions between 30 and 60% were recommended^46^. Kratz et al. (2013) found that even in 10% of the substrate, the SS harmed *Mimosa scabrella* seedlings. On the other hand, the authors observed that *Eucalyptus benthamii* production was viable on substrates containing up to 50% of the same SS^20^. These results reinforce the need to consider the characteristics of the available SS batch and the species to be produced on a case-by-case basis to define the application of this material in the substrate composition^39^.

### Recommended fertilizer rates

To reach the reference values ​​of height above 30 cm and diameter above 3 mm, the estimated CRF rates in the CS 110 days after sowing would be 3.7 and 2.5 kg m^−3^, respectively. On the other hand, considering that the minimum values ​​for both parameters were reached with a rate of 3.0 kg m^−3^ (Table 3) in the present study, this rate could be recommended. It would be necessary to apply an average of 4.8 kg m^−3^ of CRF, so the seedlings produced in the CS could reach the growth observed in seedlings grown in SS without fertilizer.

The CRF rates calculated in the present study for the CS were within the values ​​of the technical recommendations. Gonçalves et al. (2000) recommended rates between 3 and 8 kg m^−3^ of CRF (15–10-10 with micronutrients) for forest species in general^40^. Navroski et al. (2018) mentioned that recommendations could vary between 2.0 and 12.9 kg m^−3^ according to forest species and CRF formulations^14^. Davide et al. (2015) recommended rates between 3 and 5 kg m^−3^, mentioning that higher values​ can result in higher seedling growth^33^. However, the higher expenditure on CRF does not always compensate for this growth increase, and it is up to the producer to carry out this economic analysis^33^.

Other studies on *Schinus terebinthifolia* recommended CRF rates of 9.48 kg m^−3^ 13-06-16 plus micronutrients^45^, 3 kg m^−3^ 15-09-12 plus micronutrients^23^ and above 4 kg m^−3^ 15-09-12^8^. From the economic perspective, it is assumed that a rate of 3.0 kg m^−3^ of a CRF 09-15-12 with micronutrients would be adequate to produce *Schinus terebinthifolia* seedlings in the CS, reaching recommended values of height and diameter at 110 days after sowing.

It was possible to produce *Schinus terebinthifolia* seedlings using a substrate with 100% of the SS of Ilha do Governador WWTP without fertilizer application, considering a height greater than 30 cm and a diameter greater than 3 mm. The results of this and other already mentioned studies^7,19,23,39^ support that this particular SS from Ilha WWTP has solid characteristics for use as a plant substrate. Thus, the treatment of domestic sewage by activated sludge, followed by stabilization of secondary sludge by anaerobic digestion and drying for at least 90 days in deep cement beds (at least 50 cm) in full sun, generates a material suitable for use as a substrate for producing tree species seedlings. It is recommended that future studies consider evaluating the potential of SS from different WWTP, with different sludge and/or sewage treatment, to produce seedlings, as well as for other uses. It is also essential to assess which characteristics and treatments can lead to materials that are more suitable than others for a given use.

The use of SS as a substrate also implies a reduction in expenses with CS and chemical fertilizers, as verified by Ribeiro et al. (2009)^16^, Uesugi et al. (2019)^47^, Cabreira et al. (2021)^7^, and the present study. Furthermore, CRF and peat are imported to Brazil and other underdeveloped countries. These inputs are subject to fluctuation in dollar values ​​or supply crises, such as the worldwide situation experienced in 2021 and 2022, making it crucial to consider alternative materials (like the SS) with the potential to promote plants’ nutrition. Also, using SS as an agricultural input has less environmental impact than its disposal in a sanitary landfill; it can be considered more sustainable from an environmental, economic, and social point of view^10,48^. From a circular economy perspective, the transformation of waste into an input promotes the recycling of nutrients applied in food production and returns to the soil the carbon and nutrients exported from the countryside to the cities^11,21^. Future studies should generate knowledge to support the industrial production of substrates with SS^21^ and other products like organomineral fertilizers. On the other hand, extracting sphagnum peat for use as a substrate is a process that modifies and degrades natural environments^4,12^; the same can be said of mineral extraction for fertilizer production.

### Multivariate analyses

Concerning the multivariate analyses, the PCA demonstrated a solid capacity for summarizing the data, as 88.3% of the variance was represented in PC1 and PC2. The grouping of the morphological parameters revealed an affinity between the DQI and RDM, which is explained by the DQI’s formula where total dry mass (sum of the shoot and root dry mass) is a numerator. The height, diameter, H/D, and SDM formed another group of parameters. The H/D ratio and height tend to be correlated; since height is the numerator in the H/D. Also, the seedlings’ growth in height and diameter usually result in the accumulation of shoot biomass^39^, explaining the relationship between these variables.

The quality evaluation of tree seedlings’ can be subjective and even inconclusive, as a parameter should not be used alone as an indicator, but together with the others to interpret the overall quality^33^. Pondering that the PCA adjusted well to the present data, resuming seven variables into two components, the grouping of samples around the variables in the biplot can be interpreted as a complementary approach to discussing seedlings’ quality. It is crucial to consider that this interpretation of the multivariate analyses is generally in agreement with what was observed for the experimental analyzes. As mentioned above, this interpretation should not be considered separate from the previously discussed statistical analyses.

The cluster analysis made the grouping of the seedlings’ samples clearer, identifying three separate groups (Fig. 6). In the interpretation of clusters considering the seedling quality, it is possible to state that the samples in group 3 had the lowest quality. These samples showed high values for PC1, which is negatively correlated with most morphological parameters; hence these samples contain seedlings with lower values in the growth and quality variables. Groups 1 and 2 presented samples with seedlings of similar quality. Although, those in group 2 can be considered of better quality, as the samples with higher RDM are in this group, and seedlings with more roots are more likely to survive after outplanting^44^.

The substrate was the factor that best grouped the samples since observations for the SS were distributed between groups 1 and 2 (Figs. 5 and 6), with the second being formed mainly by samples of this substrate. Furthermore, observations of the CS were distributed among the three groups, with greater concentration in group 1. The CRF rate was not an important factor in grouping because it did not allow clear interpretations regarding the grouping of samples and variables. The only exception was group 3, formed by the non-fertilized seedlings from the CS.

## Conclusion

The controlled-release fertilizer rates were not significant for most variables for the SS. Without fertilizer application, the seedlings in this substrate reached the recommended values ​​for height, diameter, and quality parameters. Hence, *Schinus terebinthifolia* Raddi seedlings can be produced without fertilizers with a substrate containing sludge from the Ilha do Governador wastewater treatment plant or another plant with similar characteristics.

Regarding the commercial substrate composed of sphagnum peat (70%) and vermiculite (30%), it is recommended to apply a controlled-release fertilizer (NPK 15–09-12 with micronutrients) of 7.8 kg m^−3^ to reach the maximum growth of *Schinus terebinthifolia* Raddi seedlings, or 3.0 kg m^−3^ to reach the recommended growth values ​​for the species at 110 days after sowing.

The multivariate analyses by principal components and cluster fitted in the present data and were an adequate complementary approach to assess seedlings’ quality. Similar to what was observed in the experimental analyses, the seedlings produced in the different treatments generally presented sufficient quality for planting (groups 1 and 2), except those produced in the commercial substrate without fertilizer application (group 3).Using SS as a substrate to produce *Schinus terebinthifolia* Raddi seedlings is recommended. It can reduce nursery expenses with CS and chemical fertilizers, transforms residue into a resource, recycles nutrients used in agricultural production, and is more sustainable than SS landfill disposal.

## Supplementary Information

Supplementary Information.

## Data Availability

The datasets and R scripts generated and/or analyzed during the current study are available from JMA on reasonable request. Experimental material is not available from the authors. All authors have read and agree to the submitted version of the manuscript.
