# Supplementary material for: Sewage sludge as substrate in Schinus terebinthifolia raddi seedlings commercial production
Source: Sci Rep. 2022 Oct 14;12:17245. doi: 10.1038/s41598-022-21314-0 (PMC9568525; doi:10.1038/s41598-022-21314-0)

# **Sewage sludge as substrate in *Schinus terebinthifolia* Raddi seedlings commercial production**

Jorge Makhlouta Alonso*, Renato Nunes Pereira, Elton Luis da Silva Abel, Marjorie Ochoski, Gilsonley Lopes dos Santos, Alan Henrique Marques de Abreu.

*Corresponding Author


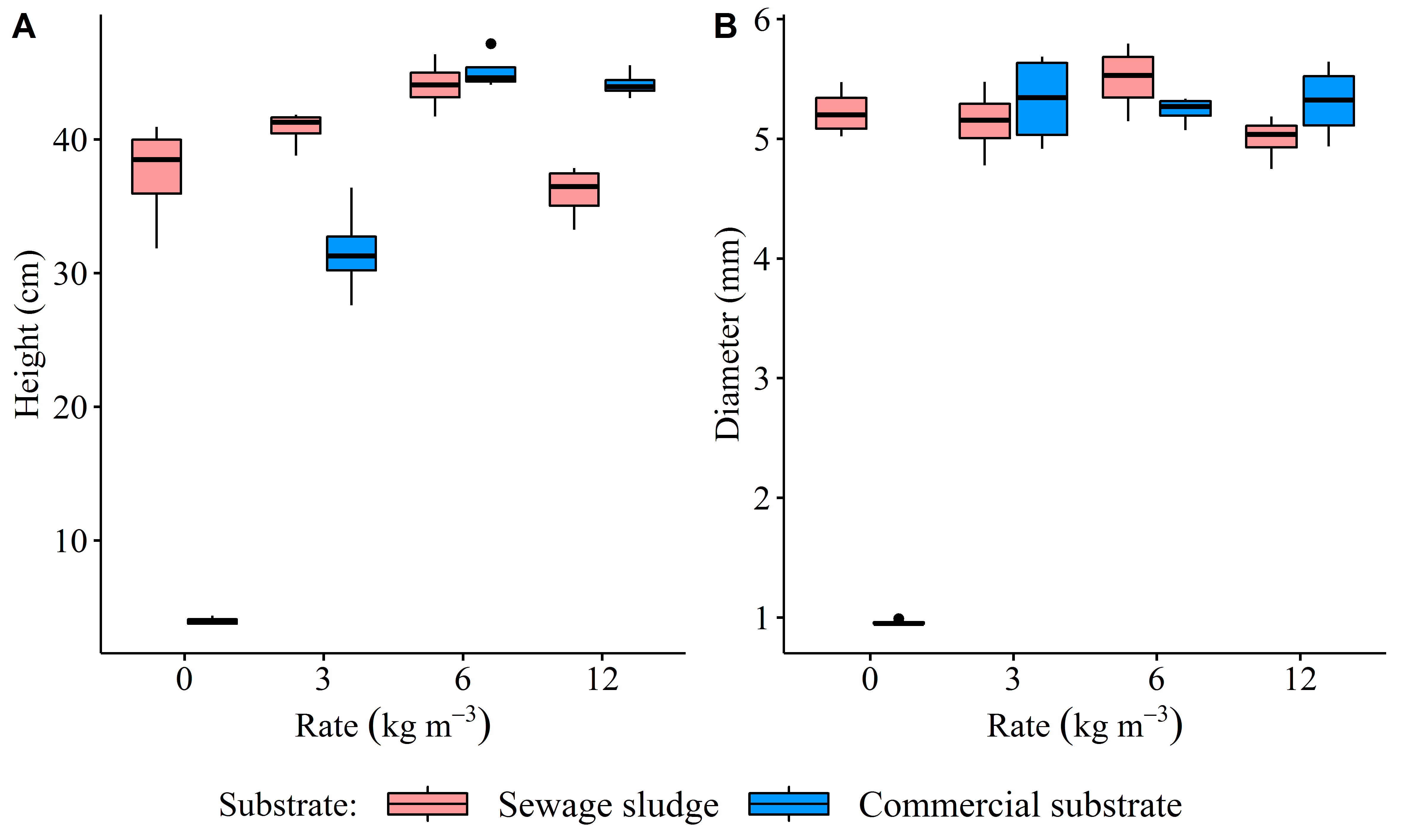


**Supplementary Fig. S1** Boxplots for (a) height and (b) diameter of *Schinus terebinthifolia* Raddi seedlings at 110 days after sowing, considering the effect of controlled-release fertilizer rates on the evaluated substrates.


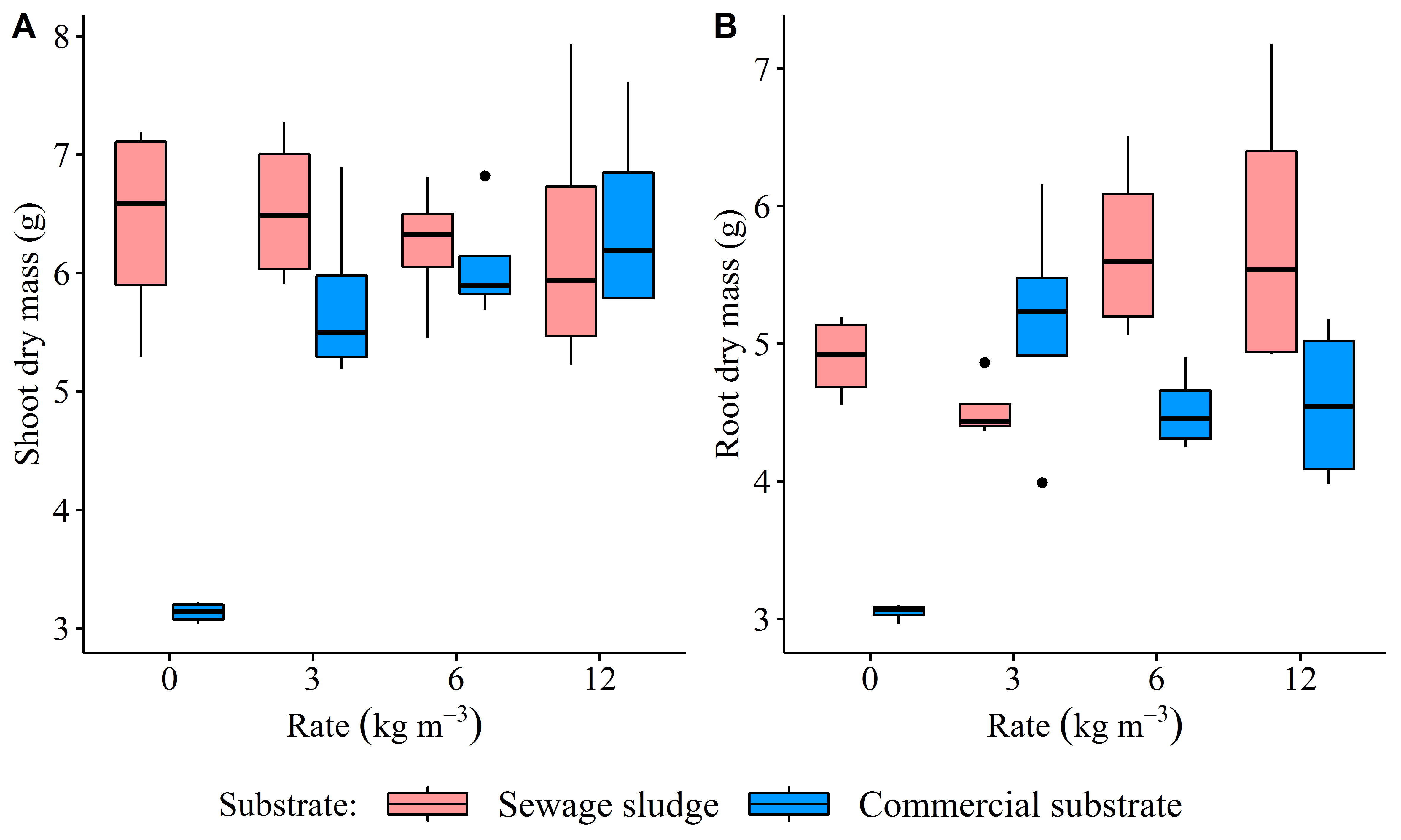


**Supplementary Fig. S2** Boxplots for (a) shoot dry mass and (b) root dry mass of *Schinus terebinthifolia* Raddi seedlings at 110 days after sowing, considering the effect of controlled-release fertilizer rates on the evaluated substrates.


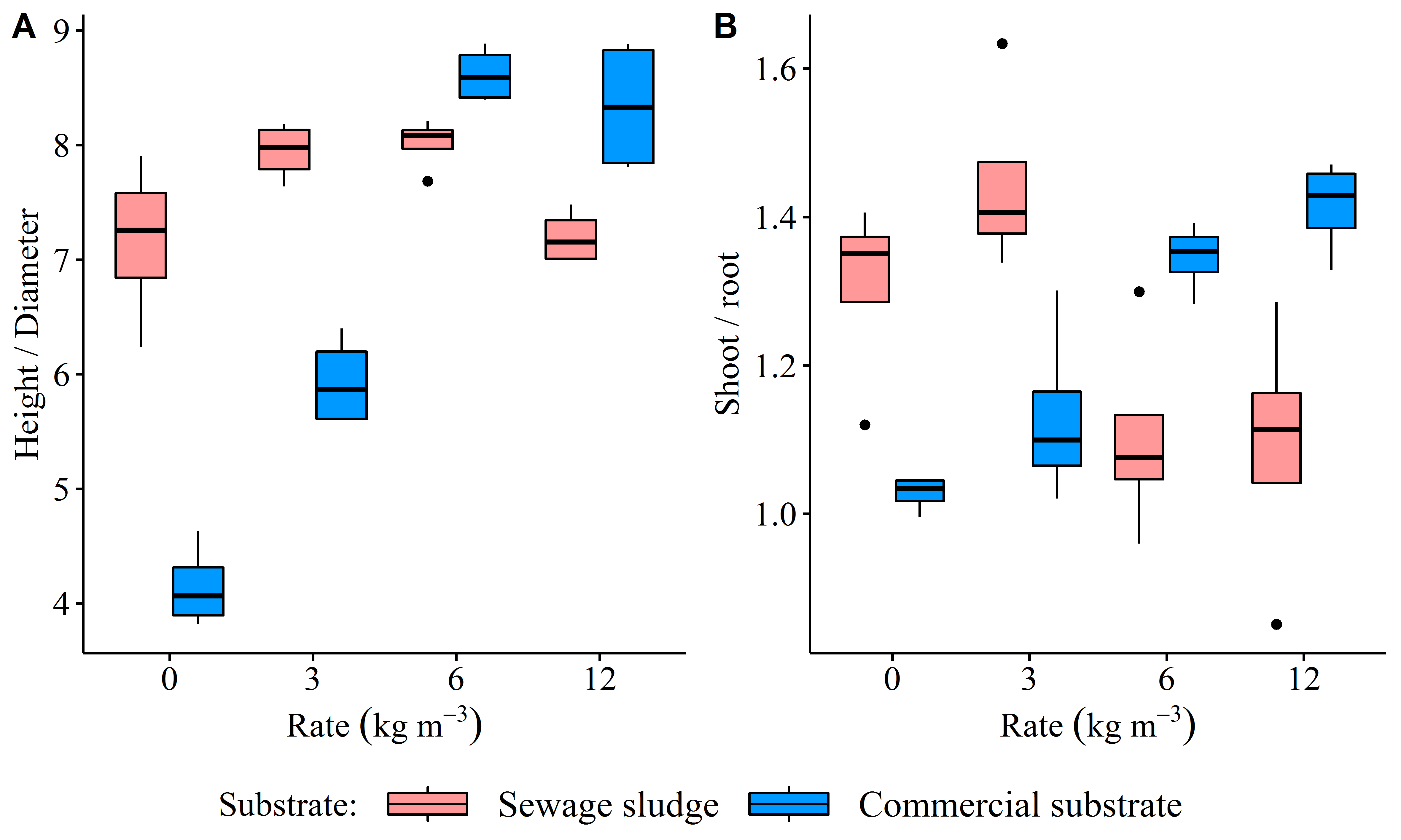


**Supplementary Fig. S3** Boxplots for (a) height/diameter ratio and (b) shoot/root ratio of *Schinus terebinthifolia* Raddi seedlings at 110 days after sowing, considering the effect of controlled-release fertilizer rates on the evaluated substrates.


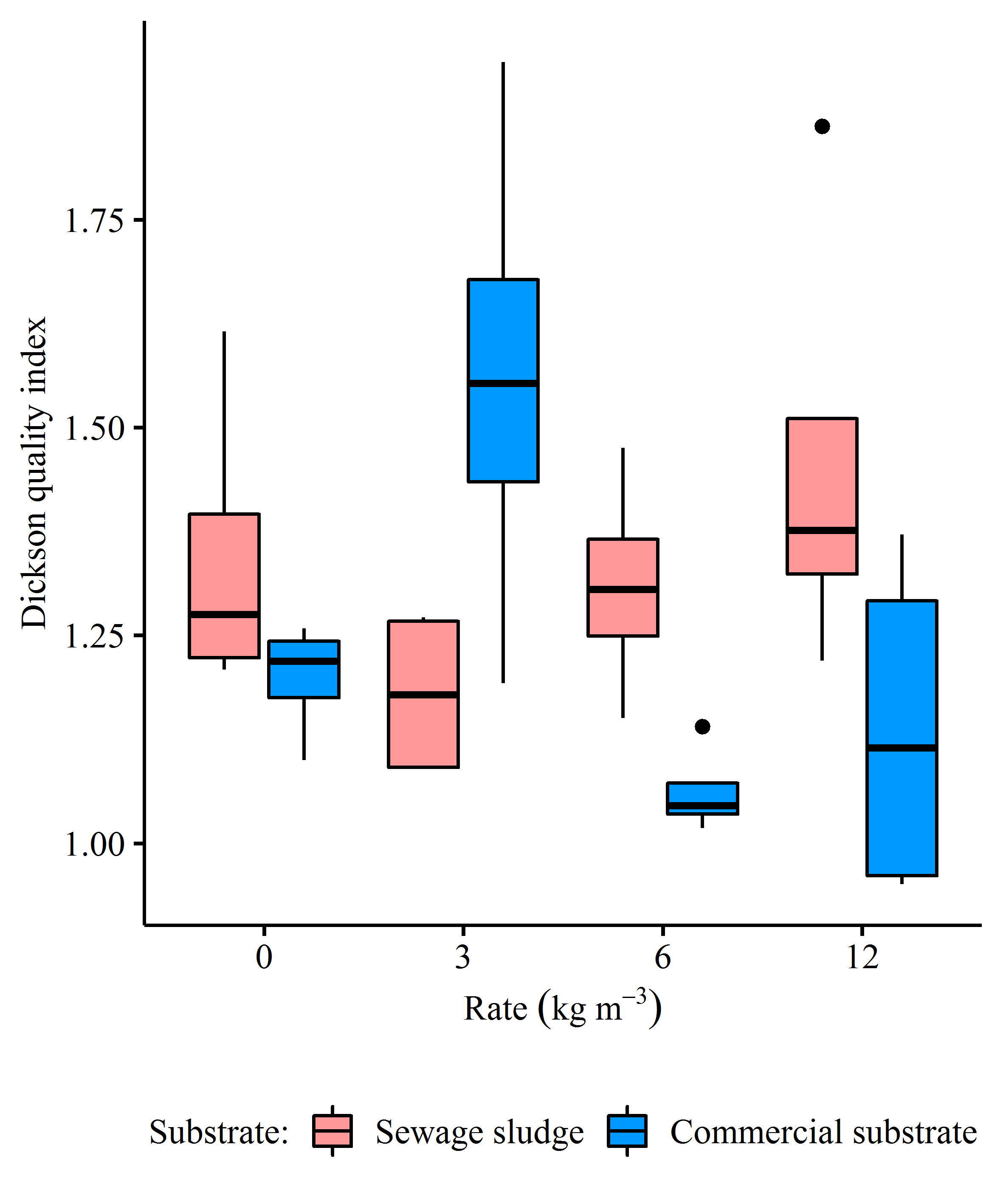


**Supplementary Fig. S4** Boxplot for the Dickson quality index of *Schinus terebinthifolia* Raddi seedlings at 110 days after sowing, considering the effect of controlled-release fertilizer rates on the evaluated substrates.


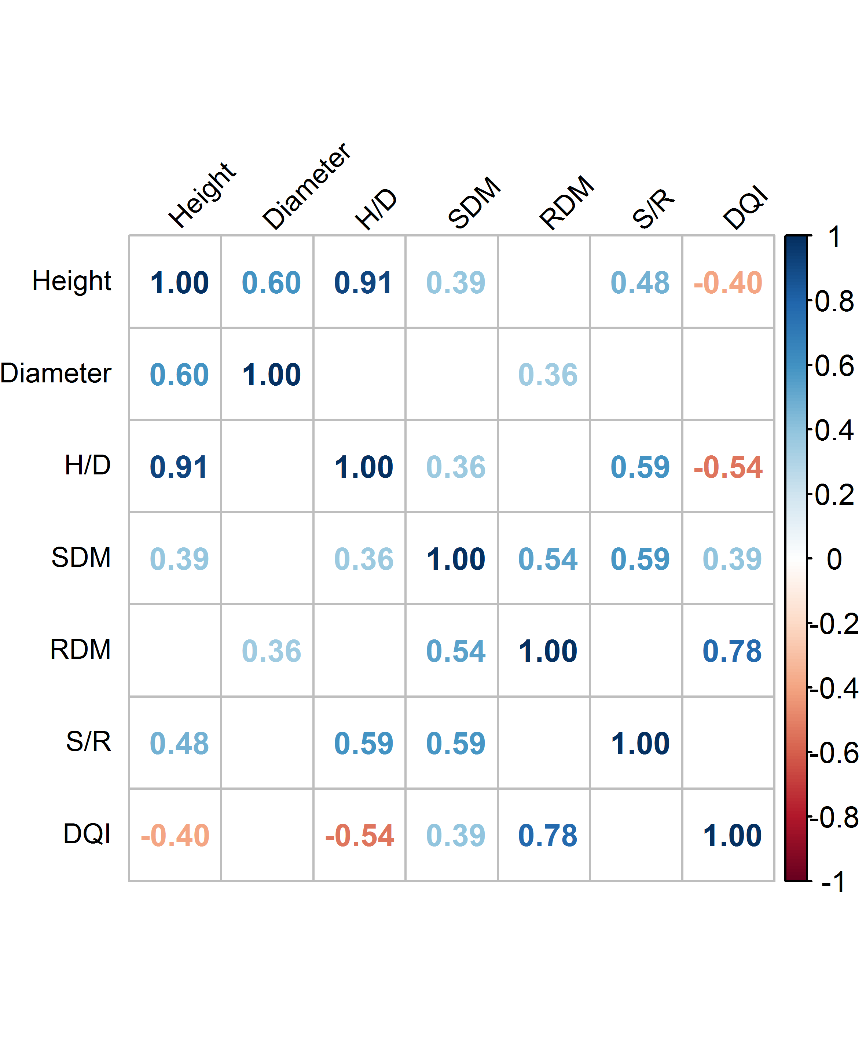


**Supplementary Fig. S5** Correlation matrix for morphological variables of *Schinus terebinthifolia* Raddi seedlings at 110 days after sowing, considering the effect of controlled-release fertilizer rates on the evaluated substrates. In which: DQI: Dickson’s quality index; H/D: height/diameter ratio; SDM: shoot dry mass; RDM: root dry mass; and S/R: shoot/root ratio.

*
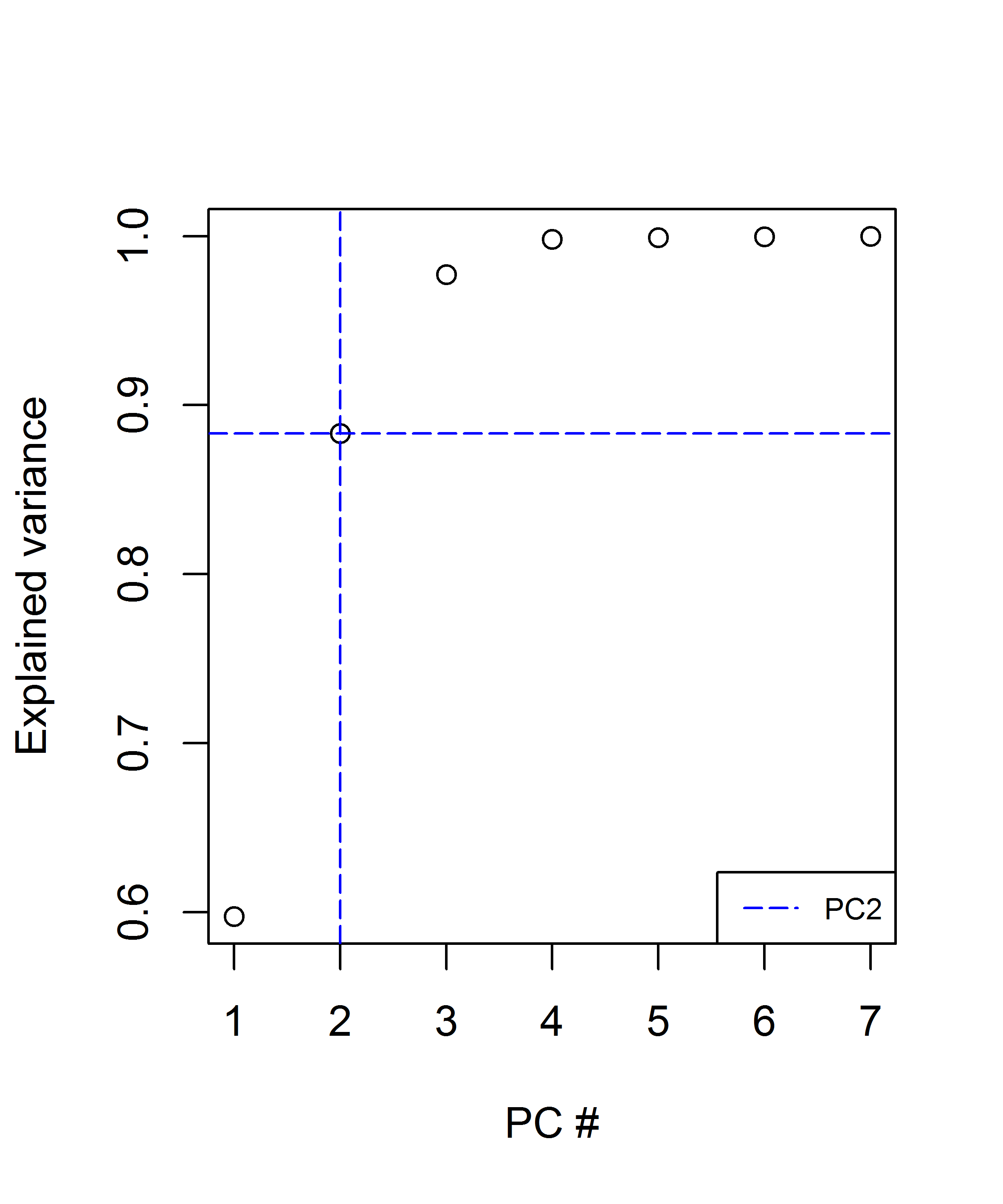
*

**Supplementary Fig. S6** Explained variance per principal components in the multivariate analysis of morphological parameters of *Schinus terebinthifolia* Raddi seedlings at 110 days after sowing, considering the effect of controlled-release fertilizer rates on the evaluated substrates.

*
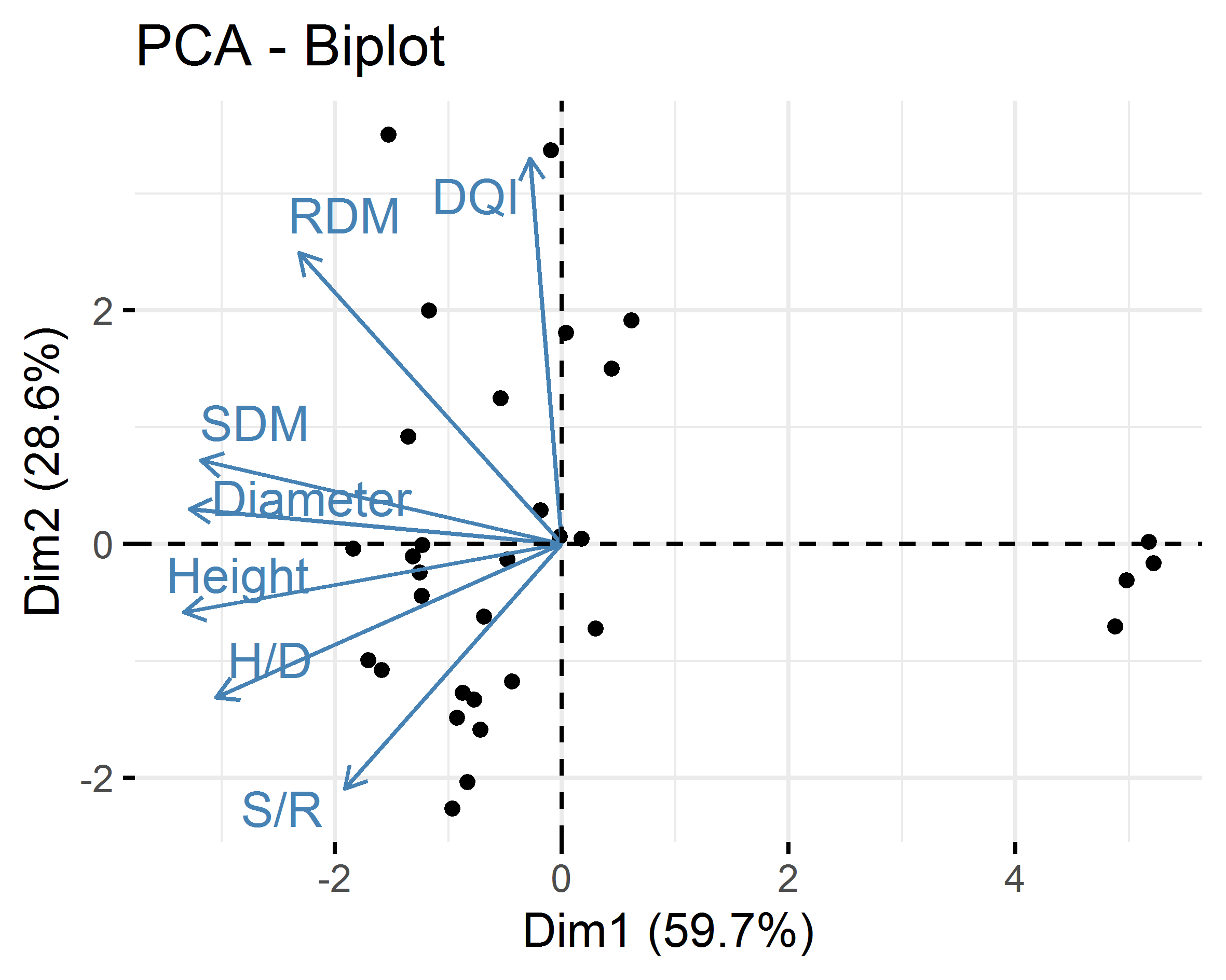
*

**Supplementary Fig. S7** Basic biplot (without aesthetic elements) of the principal components analysis for morphological parameters of *Schinus terebinthifolia* Raddi seedlings at 110 days after sowing, considering the effect of controlled-release fertilizer rates on the evaluated substrates.

*
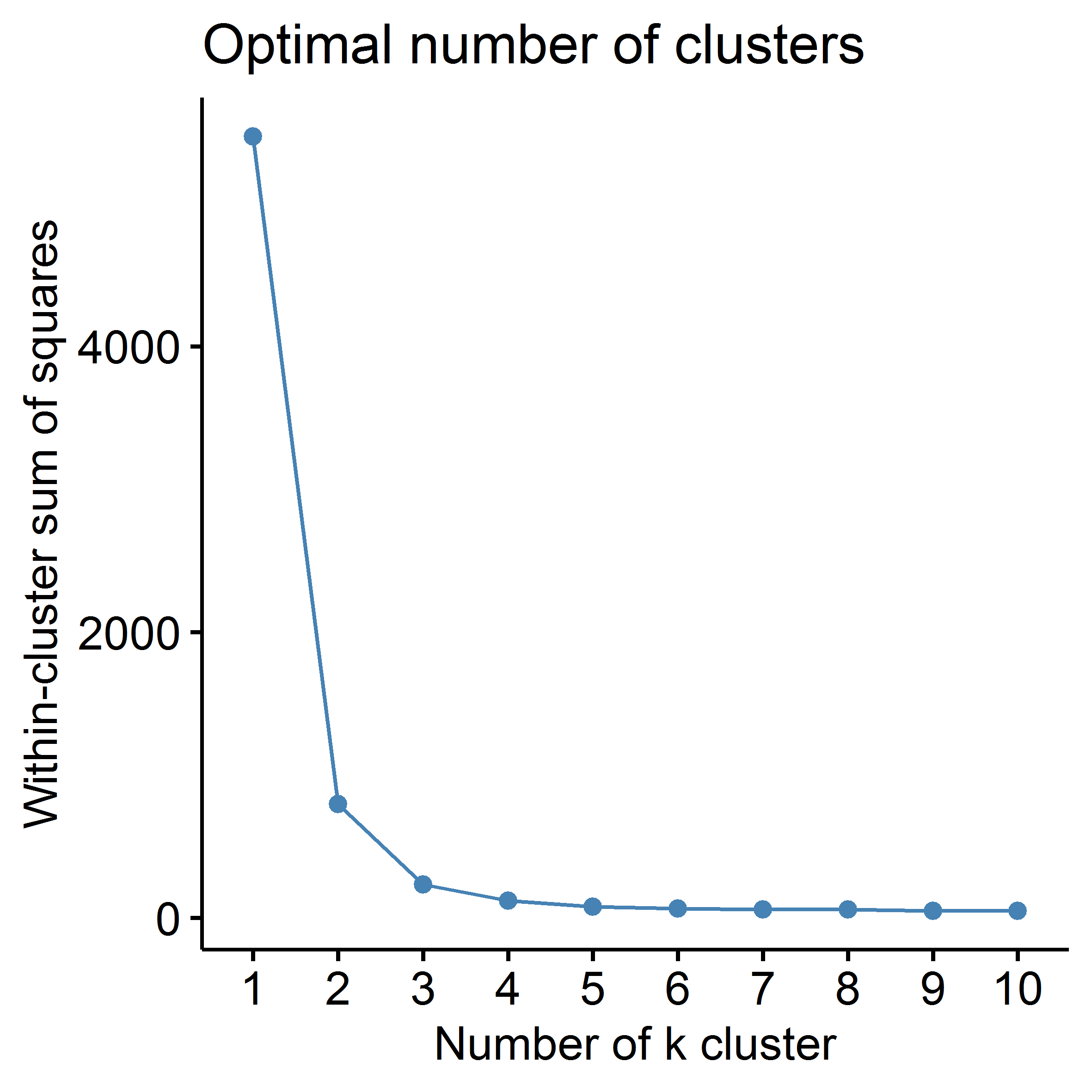
*

**Supplementary Fig. S8** Within-cluster sum of squares per cluster to identify the optimal number of clusters for morphological parameters of *Schinus terebinthifolia* Raddi seedlings at 110 days after sowing, considering the effect of controlled-release fertilizer rates on the evaluated substrates.

*
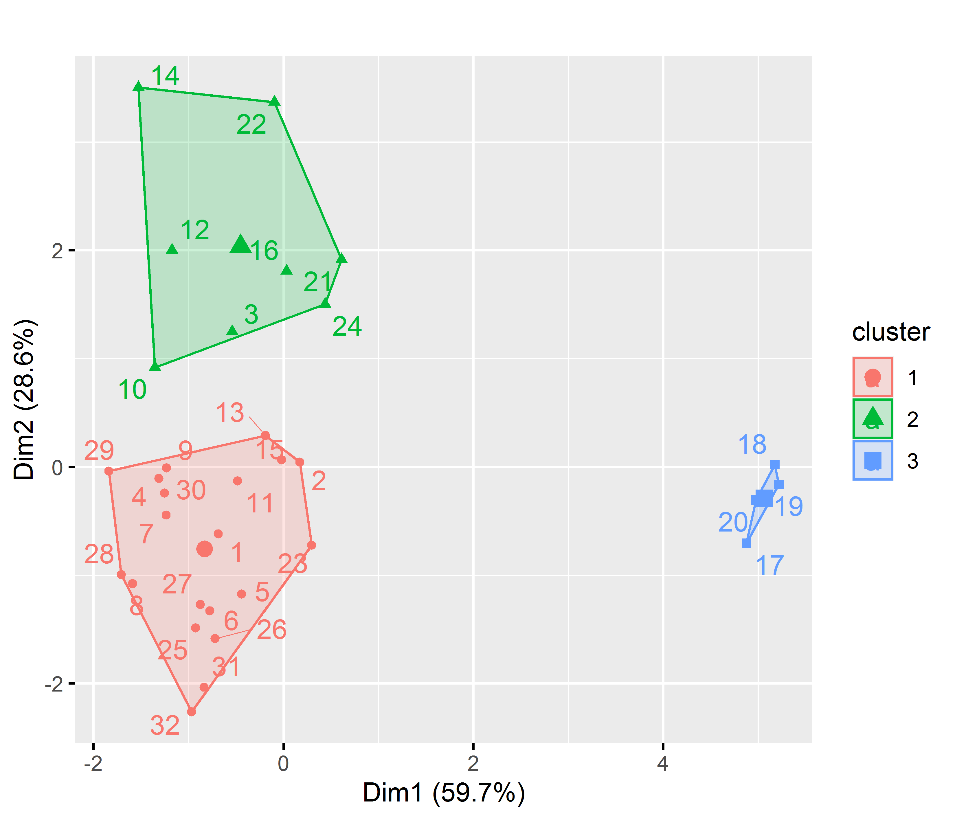
*

**Supplementary Fig. S9** Basic plot (without aesthetic elements) of the cluster analysis for morphological parameters of *Schinus terebinthifolia* Raddi seedlings at 110 days after sowing, considering the effect of controlled-release fertilizer rates on the evaluated substrates.

**Supplementary Table S10** Mean values of observations in each cluster for the morphological parameters of *Schinus terebinthifolia* Raddi seedlings at 110 days after sowing, considering the effect of controlled-release fertilizer rates on the evaluated substrates.

| **Cluster** | **Height** | **Diameter** | **H/D** | **SDM** | **RDM** | **S/R** | **DQI** |
| --- | --- | --- | --- | --- | --- | --- | --- |
| 1 | 41,51 | 5,23 | 7,95 | 6,21 | 4,65 | 1,34 | 1,17 |
| 2 | 35,80 | 5,30 | 6,72 | 6,35 | 5,95 | 1,07 | 1,59 |
| 3 | 3,96 | 0,96 | 4,14 | 3,13 | 3,05 | 1,03 | 1,20 |

In which: DQI: Dickson’s quality index; H/D: height/diameter ratio; SDM: shoot dry mass; RDM: root dry mass; and S/R: shoot/root ratio.

**Supplementary Fig. S11** Dendrogram of the hierarchical cluster analysis for morphological parameters of *Schinus terebinthifolia* Raddi seedlings at 110 days after sowing, considering the effect of controlled-release fertilizer rates on the evaluated substrates.


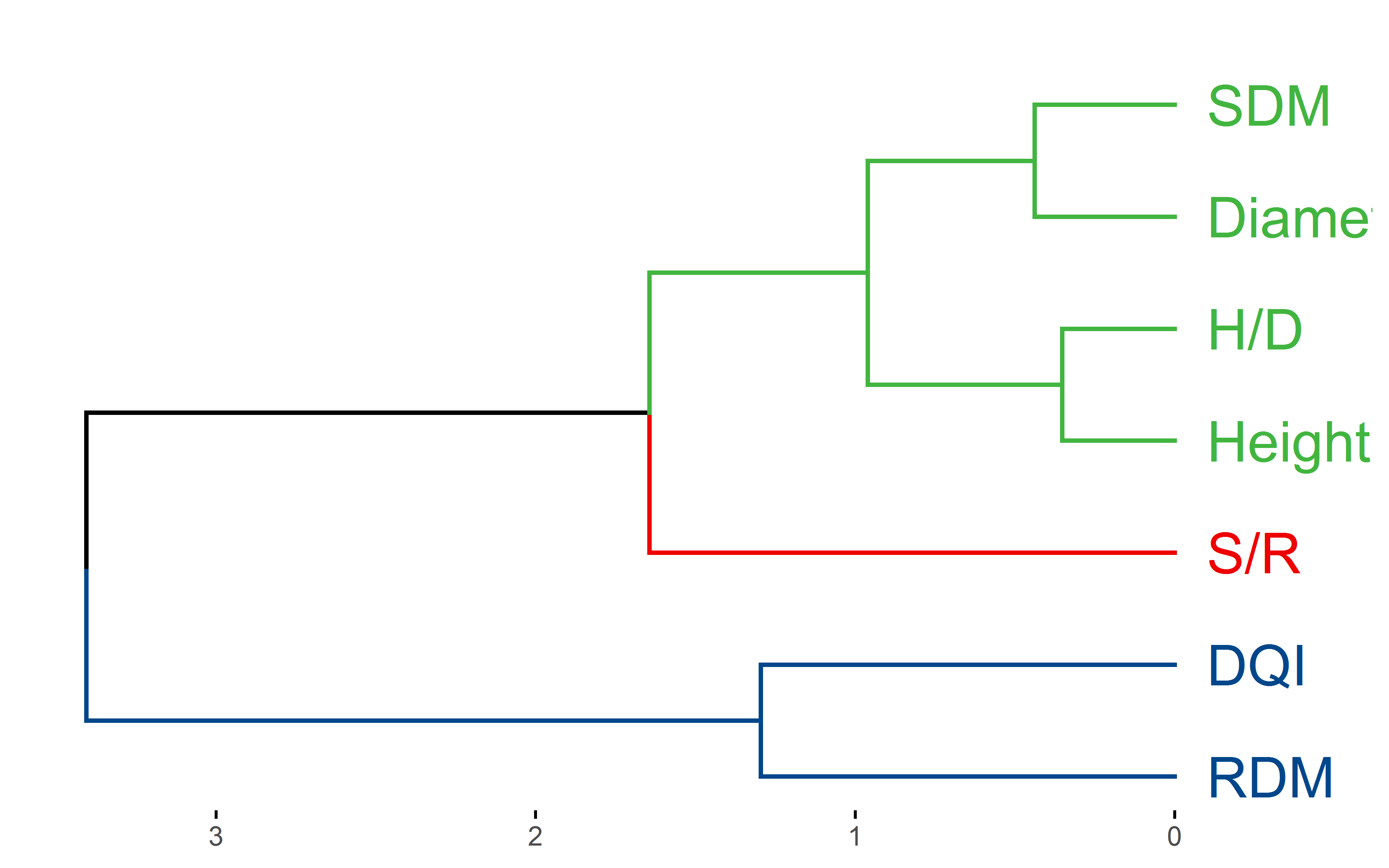

Supplement: Supplementary file 1 — Supplementary Information. [file 41598_2022_21314_MOESM1_ESM.docx]
